# Broadband large-angle beam scanning with dynamic spin energy distribution based on liquid crystal cascaded bilayer metasurface

**DOI:** 10.1515/nanoph-2023-0468

**Published:** 2023-09-29

**Authors:** Huijun Zhao, Jiayue Liu, Songlin Jiang, Xinhao Jiang, Jierong Cheng, Yunyun Ji, Shengjiang Chang, Fei Fan

**Affiliations:** Institute of Modern Optics, Nankai University, Tianjin Key Laboratory of Micro-Scale Optical Information Science and Technology, Tianjin 300350, China; Tianjin Key Laboratory of Optoelectronic Sensor and Sensing Network Technology, Tianjin 300350, China

**Keywords:** terahertz, metasurface, beam scanning, liquid crystal, spin

## Abstract

Dynamic manipulation of terahertz (THz) beams plays an important role in THz application systems. The PB metasurface provides an effective scheme for space separation and deflection of the spin beam. However, mirror symmetry locking of the conjugated spin states severely limits the versatility of the device. In this work, we demonstrate a liquid crystal (LC) cascaded bilayer metasurface that includes an LC layer, anisotropic metasurface, and PB metasurface. By controlling anisotropy and polarization conversion effects, dynamic spin asymmetric transmission is realized. Meanwhile, two different dynamic energy distribution processes are realized between the *L* and *R* state with the corresponding deflection side. The results show that the device achieves a large angular spatial dispersion within the frequency-angle scanning range of ±35° to ±75° corresponding to the broadband range of 0.6–1.1 THz. Moreover, it achieves a spin beam spatial separation with a maximum proportion of energy distribution greater than 26 dB, and the active modulation rate in the energy distribution process reaches 98 %. This work provides a dynamic THz spin conversion and efficient large-angle beam scanning, with important potentials in wavelength/polarization division multiplexing and frequency-scanning antenna for large-capacity THz wireless communication, radar, and imaging systems.

## 1 Introduction

With the development of terahertz (THz) technology, THz wavefront manipulations, especially beam deflector, plays an important role in wireless communications, imaging, and radar systems [1–3]. With the increasing demand for information transmission capacity, higher requirements are put forward for device bandwidth and channel number. In addition to wideband wavelength division multiplexing, *L* and *R*-states with opposing photonic spin angular momentum [4, 5] can further increase the number of independent channels and have unique advantages in polarization spectrum, polarization imaging, and chiral recognition. Therefore, there is a great need for spin beam deflection [6, 7], which requires a large dispersion angle range with a wide operating frequency band and the spin asymmetric transmission with a high energy distribution ratio to realize wavelength division multiplexing and spin multiplexing. However, traditional THz wavefront and polarization devices are limited due to their bulky structure and low efficiency.

Fortunately, the development of artificial metasurfaces has provided unprecedented degrees of freedom for manipulating the amplitude, phase, polarization, and wavefront of THz waves, making them have many potential applications, such as beam deflectors [8], holograms [9, 10], and Bessel beam generation [11]. In particular, the appearance of Pancharatnam–Berry (PB) metasurfaces, which are composed of half-wave plates (HWPs) with spatially varying axis, provides an effective scheme for space separation and deflection of the spin beam [10, 12–17]. For example, Zhang et al. [16] propose a reflective coded metasurface based on the PB phase to generate spin-related multi-beam scanning in free space. Wang et al. [17] fabricated the ultra-thin transmission PB metasurface, which can produce spin-related Bessel beams in the GHz frequency band. However, the mirror symmetry of pure PB metasurface makes the manipulation of a pair of conjugated spin states mutually locked and mirror symmetric.

To release the spin-locked limitations and achieve spin asymmetric deflection, the new symmetry-breaking and spatial phase distribution mechanisms are introduced into metasurface design [18–24]. For example, Yao et al. [20] proposed a bilayer metasurface-based directional device with spatially rotated micro-rods and metallic gratings, ultimately achieving asymmetric focusing. Xie et al. [24] propose a bilayer meta-atom to achieve high-efficiency dual-wavelength PB metasurfaces with independent phase control at each wavelength, which can independently achieve full 2π phase coverage at two wavelengths. However, most of these devices are passive, so once the structure is fixed, specific functions can only be implemented in a narrow frequency band.

To achieve dynamic tuning of THz beams, the most effective way is to integrate functional materials into the structural design of metasurfaces [25–27]. Among many functional materials, liquid crystal (LC) has adjustable optical anisotropy, so phase shift and polarization conversion can be actively controlled by external fields such as optical fields, temperature, electric fields, and magnetic fields, providing new opportunities for dynamic manipulation of THz waves [28–33]. Recently, Zhuang et al. [32]. introduced a metasurface with LC elastomer and applied line-focused infrared light to manipulate broadband wavefront steering. Naveed et al. [34] employed LC-integrated spin-decoupling metasurfaces to change the holographic image from a “Sun” to a “Star”. However, in the THz band, the tunable polarization conversion and spin chirality brought about by the integration of the broadband anisotropy of the LC with the metasurface have not been fully utilized in terms of beam scanning, spin selection, and energy distribution.

**Figure 1:**
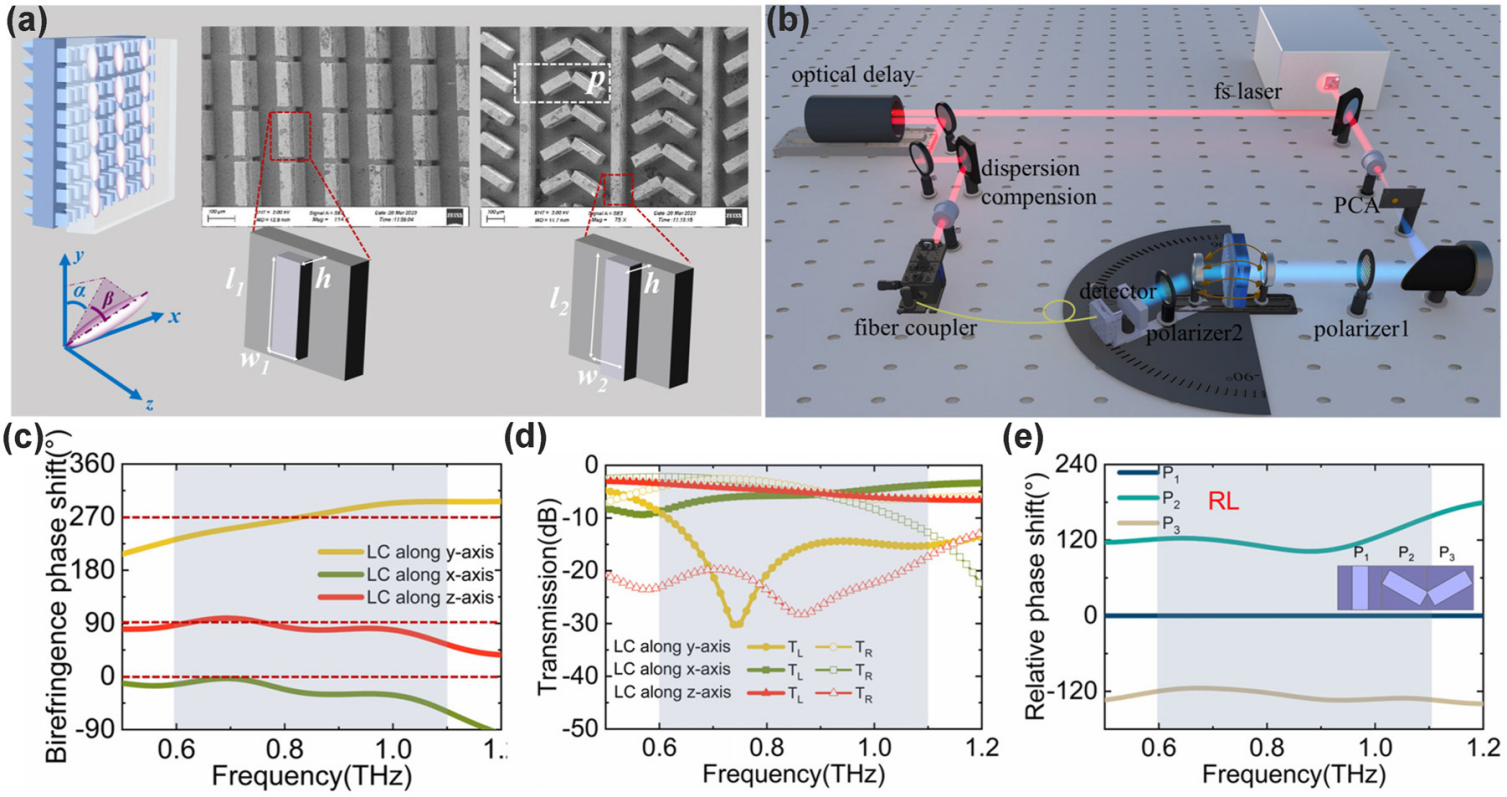
Device structure and design. (a) Geometry of the metadevice and SEM photos of the two metasurfaces; (b) schematic diagram of the AR-THz-TDPS system; (c) the simulated birefringence phase shift between the *x*- and *y*-directions through the LC-anisotropic metasurface; (d) the simulated transmission spectra of *L* and *R* spin states through the LC-anisotropic metasurface when the +45°-LP wave input. (e) Relative phase spectra of spin-flip state *RL* for the three meta-atoms of the PB metasurface layer (*P*_1_, *P*_2_, and *P*_3_).

In this paper, we show an LC-integrated metadevice that cascaded an LC layer, an anisotropic metasurface layer, and a PB metasurface layer, as shown in Figure 1. By designing the tunable birefringence phase shift and chirality combined with the LC layer and the anisotropic metasurface, the dynamic anisotropy and spin conversion effect are realized. When the beam is finally deflected out of the PB metasurface, a dynamic spin asymmetric transmission is obtained due to precisely changing spatial phase distributions. Meanwhile, based on the different orientations of LC molecules (*y*-to-*z* and *y*-to-*x*) under the external field, two different dynamic energy distribution processes are realized between the two sides of the deflection angles corresponding to the left and right-rotated spin states (i.e. the *L* and *R* states). The device achieves a large angular spatial dispersion within the range of ±35° to ±75° corresponding to the broadband operating range of 0.6–1.1 THz.

## 2 Methods

### 2.1 Device structure and fabrication

The proposed LC integrated bilayer metasurface is shown in Figure 1(a), of which structure is as follows: (1) the top glass substrate with a pre-oriented anchor layer in the *y*-direction; (2) LC layer; (3) anisotropic metasurface, which is the front layer of the bilayer Si metasurface and also has a pre-oriented layer in the *y*-direction on its surface; and (4) PB metasurface, which is the back layer of the bilayer Si metasurface. These layers are encapsulated to an integrated metadevice by the ultraviolet glue. To homogeneously pre-oriented LC molecules along the *y*-axis, two polyimide (PI) films are coated on the JGS1 glass substrate and the surface structures of the metasurface, and then mechanically rubbed separately, resulting in micro-nanoscale grooves and molecular chains on the surface of the anchor layer, so this process makes the LC molecule chains aligned along the friction direction (the *y*-axis). The bilayer metasurface was fabricated on a high resistance Si substrate of >10 KΩ cm and 1 mm thickness by the processing of the photolithography and reactive ion beam etching with the etching depth of *h* = 200 μm and the meta-atom period of *P* = 180 µm for both front and back sides. The anisotropic metasurface consists of etched rectangular columns with a length of *l*_1_ = 160 μm and a width of *w*_1_ = 50 μm. The PB metasurface is composed of discrete axis-orientation atoms with a length of *l*_2_ = 180 μm and a width of *w*_2_ = 60 μm. The long axis direction of the rectangular column is oriented at a different angle in the substrate plane, *θ* = 0°, 60°, and 120° for the three meta-atoms, respectively. As shown in Figure 1(a), the meta-atoms of *θ* = 0° are connected end to end along the *y* direction as a grating structure, and a pair of the adjacent 60° and 120° meta-atoms are joined together at one corner of the rectangle.

The LC used in this work is a high birefringence LC (HTD028200) from Jiangsu Hecheng Technology Co., Ltd., its extraordinary and ordinary refractive indices (*n*_*e*_ and *n*_*o*_) are 1.9 and 1.6 in the THz regime, respectively, so its birefringence is Δ*n* = 0.3. The total thickness of the LC layer in the metadevice is 700 μm including the portion filled into the rectangular column gap. Because the column spacing is on the order of ten microns, the size of the LC molecules is much smaller, and the direction of the LCs inside the Si column gap is the same as that outside the column. Therefore, the interaction between LC molecules and Si columns is negligible in the following discussions. A pair of hollow electromagnets applies a tunable external magnetic field (EMF) from 0 to 60 mT to the metadevice in the *x* and *z* directions, respectively, so that the original LC molecular arrangement is disrupted. When the maximum EMF is applied, it will be evenly arranged in the direction of the EMF. We define the angle between the LC principal axis and the *x*–*y* plane as *β*, and the angle between the projection of the LC main axis on the *x*–*y* plane and the *y*-axis as *α*. The following work discusses device performance due to tunable birefringence phase shift caused by the LC layer and the anisotropic metasurface under different LC molecular orientation angles *α* and *β*. Based on previous research [35–37], it can be estimated that the response time of the device is on the order of ten to hundred milliseconds, while the switching transition time may be affected by the EMF, anchoring layer, and the viscosity coefficient of the LC material.

### 2.2 Simulation design

First, we simulated the tunable birefringence phase shift of the LC layer and the anisotropic metasurface in the orthogonal directions along the *x*, *y*, and *z*-axis in Figure 1(c). The anisotropic metasurface design has three main aims: (1) it provides an additional anisotropic phase shift, thus changing the tunable phase shift range of the LC; (2) by matching the anisotropy axis of the metasurface with that of the liquid crystal, the spatial mirror symmetry of the device is broken, and the optical chirality of the whole device is obtained; (3) a negative dispersion phase shift of the anisotropic metasurface is obtained by structural design, and the dispersion can be offset with the positive dispersion phase shift of the LC layer, so a near-zero dispersion phase shift is obtained in a broadband range.

The simulation method can be found in Section S3 of Supplementary Material. When the EMF is 0 T, we set the LC along the *y*-axis under the role of the surface orientation layer, the birefringence phase shift value is about 270° as the yellow line in Figure 1(c). Therefore, when 45°-linear polarized (LP) light is incident on this composite LC metasurface, the output is mainly the *L*-spin component, as shown by the yellow line in Figure 1(d). When a longitudinal EMF is applied and the LC is rotated along the *z*-axis, as shown by the orange lines, the birefringence phase shift is about 90°, and the 45°-LP light is mainly converted to the *R*-spin state. When a transverse EMF is applied and the LC is tuned along the *x*-axis, the birefringence phase shift is around 0°, and there are both *L* and *R* states in the outgoing beam. As can be seen in Figure 1(c), the phase shift curves of the LC-integrated anisotropic metasurface are flat, and the phase shift dispersion is well eliminated in the broadband range by the structural design. Therefore, by designing the tunable birefringence phase shift combined with the LC layer and the anisotropic metasurface, the dynamic anisotropy and spin conversion effect are realized in the broadband range of 0.6–1.1 THz.

For the PB metasurface layer, the PB meta-atom is designed as a half wave-plate (HWP) with a birefringence phase shift of 180° also in the broadband range of over 0.6–1.1 THz in Section S4 of Supplementary Material. The input spin states will be converted to their spin-flip states (input *L*-output *R* state, marked as *RL* state; input *R*-output *L* state, marked as *LR* state). In addition, each meta-atom will introduce a geometric phase shift of 2*φ*, where *φ* is the rotation angle of PB meta-atoms. We simulated the phase spectra of different PB meta-atoms *P*_*n*_ (*n* = 1, 2, and 3) relative to *P*_1_, that is Δ = *P*_*n*_ − *P*_1_, as shown in Figure 1(e), using the *RL* state as an example. There is a negative spatial gradient phase distribution in the turn of *P*_1_ < *P*_2_ < *P*_3_ with a step of 120° in the whole frequency range of over 0.6–1.1 THz, and finally the total phase shift is 2π in one PB supercell. Therefore, in the case of *L*-state incidence, the *R*-state will be deflected to the right side (defined as a negative deflection angle). As shown in Figure S1 of Supplementary Material, when the *R*-state is incident on this PB metasurface, the *LR* state will be deflected to the left side (defined as the positive deflection angle). The scanning deflection angle *θ* of the outgoing beam and the frequency *f* meet the following phase-matching relation: sin *θ* = ±*c*/(3*Pf*), where *P* = 180 μm and *f* is over 0.6–1.1 THz, so the corresponding scanning angle range is over 30–70°.

## 3 Results and discussion

### 3.1 Polarization and chirality modulation processes

**Figure 2:**
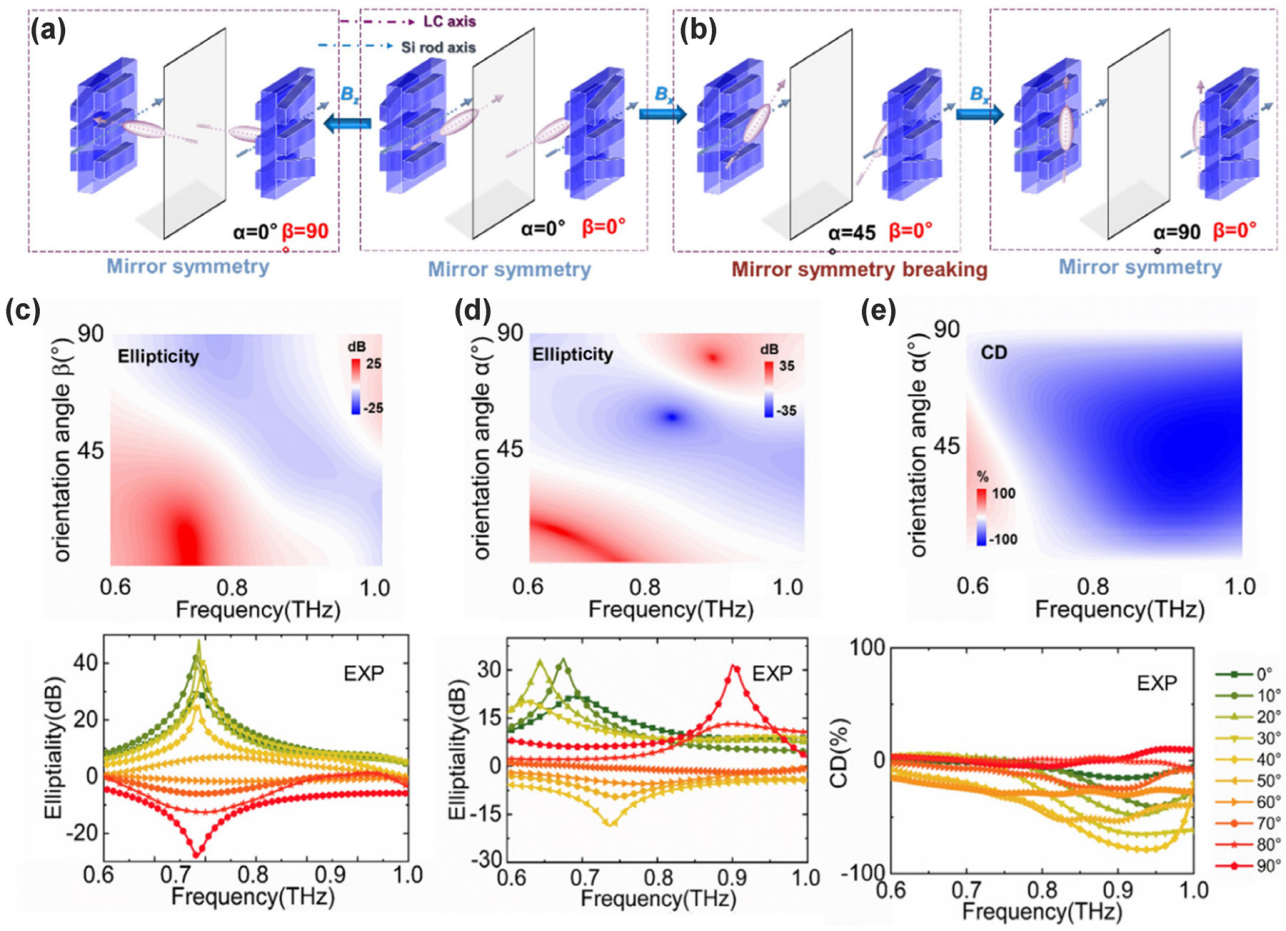
Geometric symmetry of metadevice with (a) LC rotated in the *y*–*z* plane and (b) in the *x*–*y* plane; (c) the simulated and experimental ellipticity spectra of the LC-integrated metadevice varying with the orientation angle *β* from 0 to 90° (*α* = 0°) and the frequency under an increasing longitudinal magnetic field; (d) the ellipticity spectra and (e) CD spectra maps varying with the orientation angle *α* from 0 to 90° (*β* = 0°) and frequency under an increasing transverse magnetic field.

First, we are concerned with the dynamic anisotropy and chirality of the whole device under external field manipulation. The anisotropy is determined by both the LC orientation and the structural orientation of the metasurface, so as long as the LC orientation changes, the output polarization state will change, but the corresponding results are different when the *α* and *β* angles change. If one of *α* and *β* is always 0 (i.e. the LC orientation is always confined to rotation in the *x*–*y* or *y*–*z* planes), then the case for chirality becomes relatively simple. As shown in Figure 2(a), when *α* = 0° and *β* changes in the range of [0°, 90°], the transmission system has mirror symmetry along the propagation direction (*z*-axis), so the device has only a dynamic anisotropy but no chirality in this case. When *α* changes in the range of (0°, 90°) and *β* = 0° as shown in Figure 2(b), there is an angle between the optical axis of LC and the optical axis of the anisotropic metasurface but not in the same spatial plane, where the mirror symmetry is broken. In this case, the device has both tunable anisotropy and chirality, with the strongest chirality occurring at *α* = 45°.

The above processes on the polarization state and chirality are demonstrated in the normally outgoing signals (*θ* = 0°) by the simulation and experiments. We discuss the ellipticity of the metadevice with the EMF and frequency, which follows the intensity difference between the *L* and *R* spin components of the outgoing light. As shown in Figure 2(c), when LC orientation rotates in the *y*–*z* plane under the longitudinal EMF, the ellipticity gradually decreases, and the exit polarization state gradually converts from *L* to *R*-state. When LC orientation rotates in the *x*–*y* plane under the transverse EMF, the ellipticity decreases from a positive peak to a negative value in a lower frequency band and then increases to another positive peak again at a higher frequency band, as shown in Figure 2(d).

In the same case, the CD spectra are also obtained as shown in Figure 2(e). The CD increases from 0 to −90 % in the range of 0.6–1.0 THz as the LC orientation *α* rotates from 0° (along the *y*-axis) to 45° in the *x*–*y* plane. When the LC orientation continues to rotate to the *x*-axis, the CD value drops from the maximum to 0. It is worth noting that whether there is chirality means whether the spin transformation and the transfer are symmetric. Obviously, in the first case (LC rotates in the *y*–*z* plane), the chirality is 0 in the whole process, so the polarization conversion is always symmetric for *L* and *R* spin states; In the latter case (LC rotates in the *x*–*y* plane), the chirality of the intermediate process is not 0, in which case the transmission property and polarization conversion are asymmetric.

### 3.2 Spin conversion and energy distribution in different magnetic field directions

**Figure 3:**
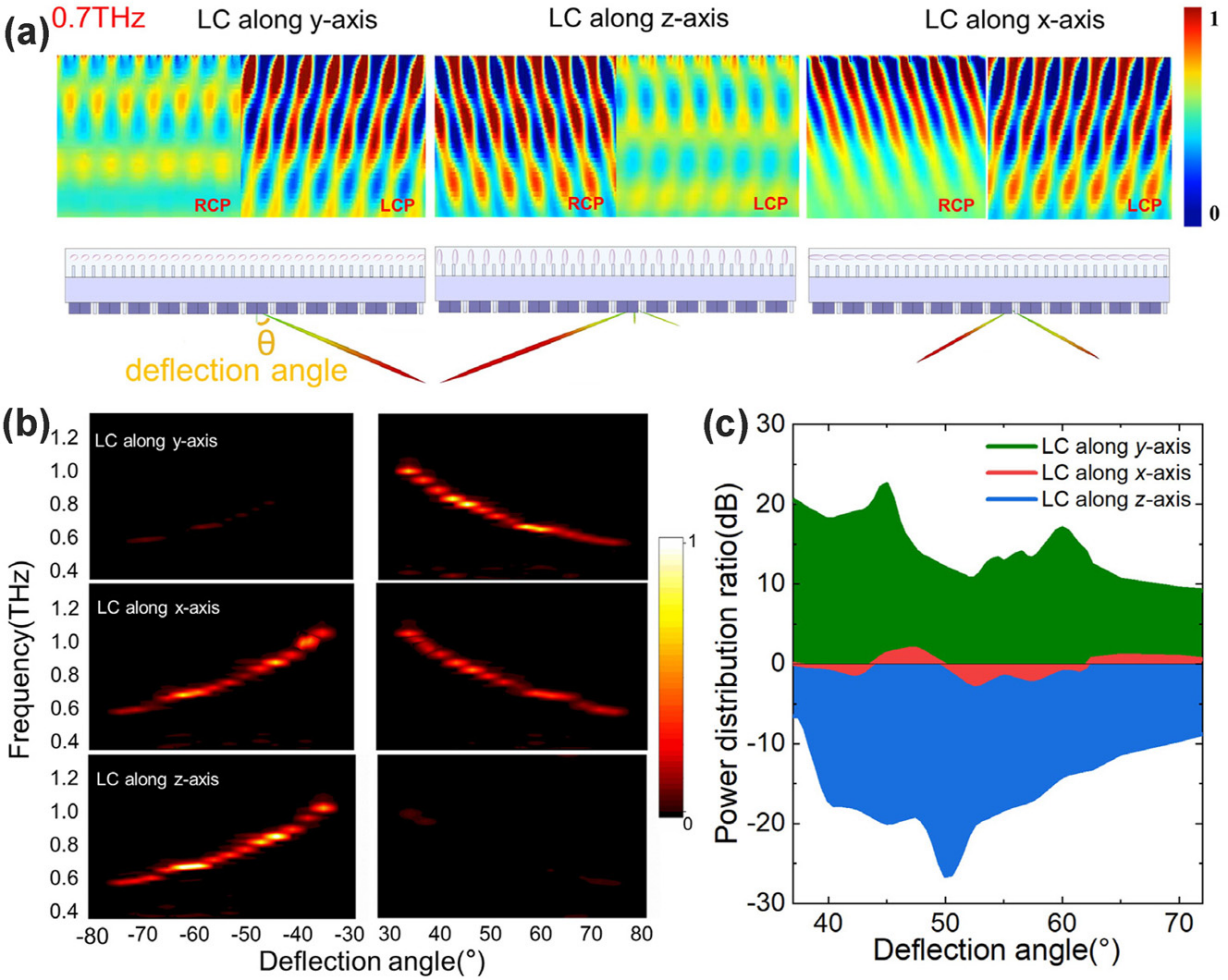
Beam scan results. (a) The simulated electric field distributions and far-field scattering patterns of different spin states at 0.7 THz with different LC orientations when 45°-LP light is incident. (b) Experimental THz diffraction efficiency and (c) energy distribution ratio between *L* and *R* spin states through the LC integrated metadevice at different deflection angles *θ* with LC orientations along *x*, *y*, and *z* axis under the different EMF directions.

Based on the dynamic anisotropy and spin conversion mechanism of the composite anisotropic metasurface, combined with the beam splitting effect of PB metasurface on different spin states, the spin asymmetric scanning is realized. Figure 3(a) shows the electric field distributions and far-field patterns of different spin states through the metadevice under different LC orientations at 0.7 THz by the FDTD simulation. When there is no EMF applied and the LC molecules are along the *y*-axis, the outgoing beam is the *L*-state and deflected to the right side (i.e. *θ* > 0). When a longitudinal EMF is applied and the LC is orientated along the *z*-axis, the outgoing beam is the *R*-state and deflected to the left side (i.e. *θ* < 0). After applying a transverse EMF, the LC molecules turn to the *x*-axis direction, and the outgoing *L* and *R* states are equally distributed to the right and left deflection. Since the PB metasurface satisfies the phase matching condition, it can spatially separate THz spin states dependent on the frequencies, and for the two conjugated spin states, they are always deflected in different directions, so this active metadevice can realize both wideband wavelength and spin division multiplexing.

Next, the experiments for two spin states through the metadevice at different deflection angles have been done by the angle-resolved THz time-domain polarization spectroscopy (AR-THz-TDPS) system. The THz polarizer in front of the sample is fixed at 45° with the *x*-axis and the polarizer behind the sample is rotated along the *y*-axis. THz signals are detected by rotating the THz detector in different directions and angles. If we take the intensity transmission of air without samples as a reference to obtain the intensity transmittance of the metadevice, the peak transmittance can reach 52 % at the deflection angle of 42.5°. Considering the insertion loss due to interface reflection and material absorption, the device has high diffraction efficiency. If the factor of interface impedance is removed, the diffraction efficiency of the device at this frequency and deflection angle is close to 100 % when we use the bare glass-LC-Si cell without metasurface as a reference. Therefore, we normalize all the experimental data as this peak value to obtain the normalized intensity transmittance in the following results. As shown in Figure 3(b), when no EMF is applied and the LC orientation is along the *y*-axis, significant signals can be detected between the deflection angle *θ* of 35°–75°, of which the corresponding frequency range is 0.6–1.1 THz. However, the intensity transmittance at *θ* < 0 is negligible, so there is no defection to the left side, and the detailed data of the intensity spectra are presented in Section S2 of Supplementary Material. When a 60 mT transverse EMF is applied, the right and left side diffraction energy is equally distributed. When a longitudinal EMF of 60 mT is applied, a valid signal can be detected at the left side with deflection angles of −35° to −75°.

To characterize the performance of the energy distribution, we calculate the energy distribution ratio (*R*_ED_) as follows: *R*_ED_(*θ*) = 10log[*I*_*+*_(*θ*)/*I*_−_(*θ*)], where *I*_+_ and *I*_−_ are the intensity transmittance at positive and negative deflection angles, as shown in Figure 3(c). When *R*_ED_ is positive, it indicates that the beam is deflected towards a positive angle, while when *R*_ED_ is negative, it indicates that the beam is deflected towards a negative angle. When the LC orientation is along the *y*-axis, *R*_ED_ > 10 dB, and the maximum can reach 23.5 dB. When the LC orientation is along the *z*-axis, *R*_ED_ > −10 dB (a negative value means more energy to the left), and the maximum proportion of energy distribution when the energy is distributed on the left and right sides can reach −26 dB. When a transverse EMF is applied, the *R*_ED_ is around 0, indicating that the energy is equally distributed for the two sides.

**Figure 4:**
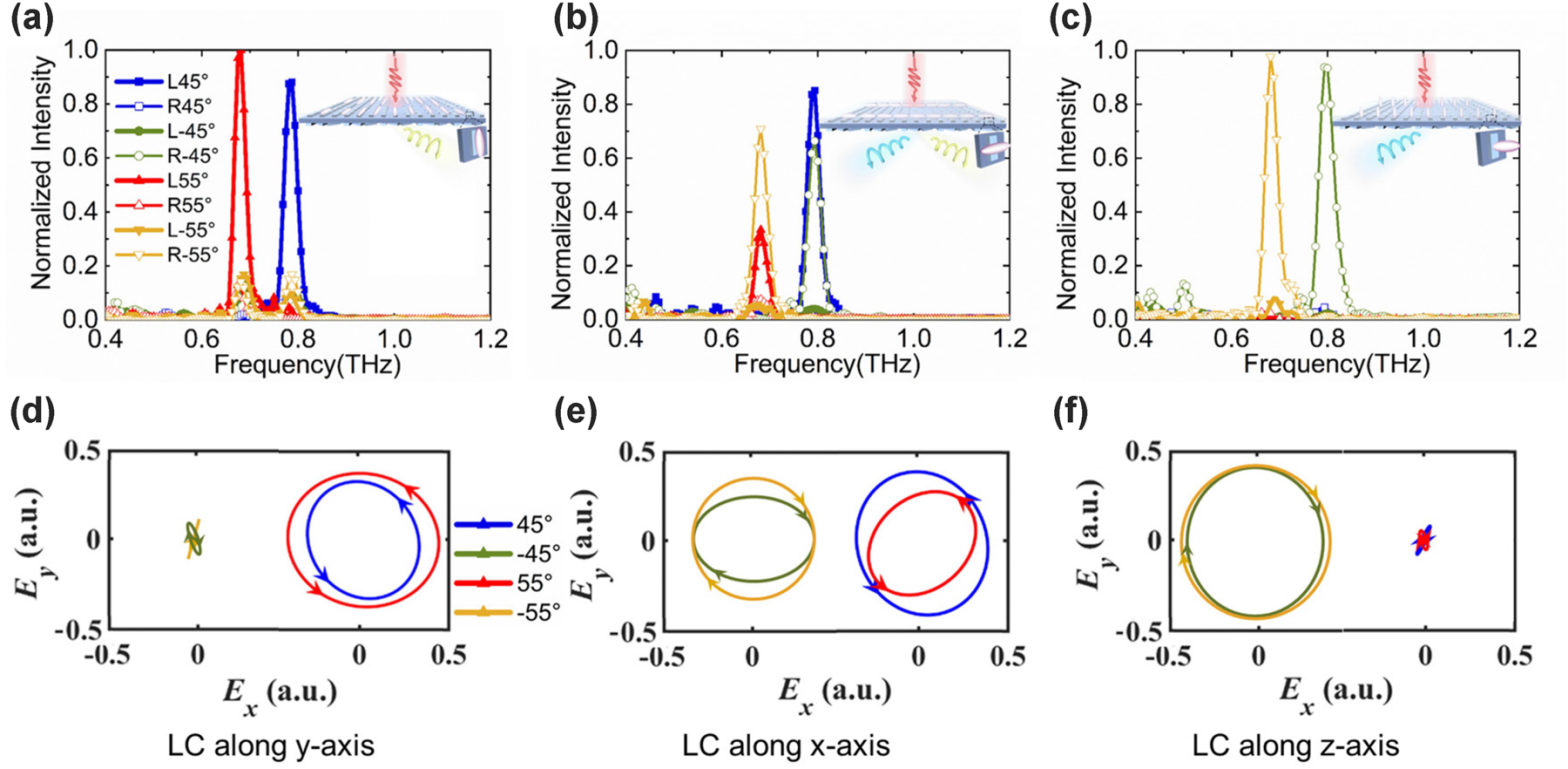
Experimental transmission spectra of different spin states at deflection angles *θ* of ±45° and ±55° under different EMF directions: (a) no EMF; (b) EMF is along the *x*-axis; (c) EMF is along the *z*-axis. The corresponding polarization ellipses with (d) LC orientation along the *y*-axis; (e) the *x*-axis; (f) the *z*-axis.

To further confirm the polarization state of the deflected signal, we can rotate the second THz polarizer to ±45° to measure the ±45° LP components, and then obtain the transmission spectra of the *L* and *R*-states, also further plot the polarization ellipse according to Section S1 of Supplementary Material. As shown in Figure 4, only take *θ* = ±45° and ±55° as examples. The experiments demonstrate that no matter how the energy is distributed, the deflection wave to the left side is always *R*-state, and the deflection wave to the right is always *L*-state, which is completely consistent with the above analysis and simulations. Therefore, based on the dynamic anisotropy mechanism, this metadevice realizes active spin conversion and excellent energy distribution in the broadband working region of 0.6–1.1 THz, with the frequency-scanning range of ±35° to ±75°.

### 3.3 Dynamic energy distribution with a longitudinal magnetic field

**Figure 5:**
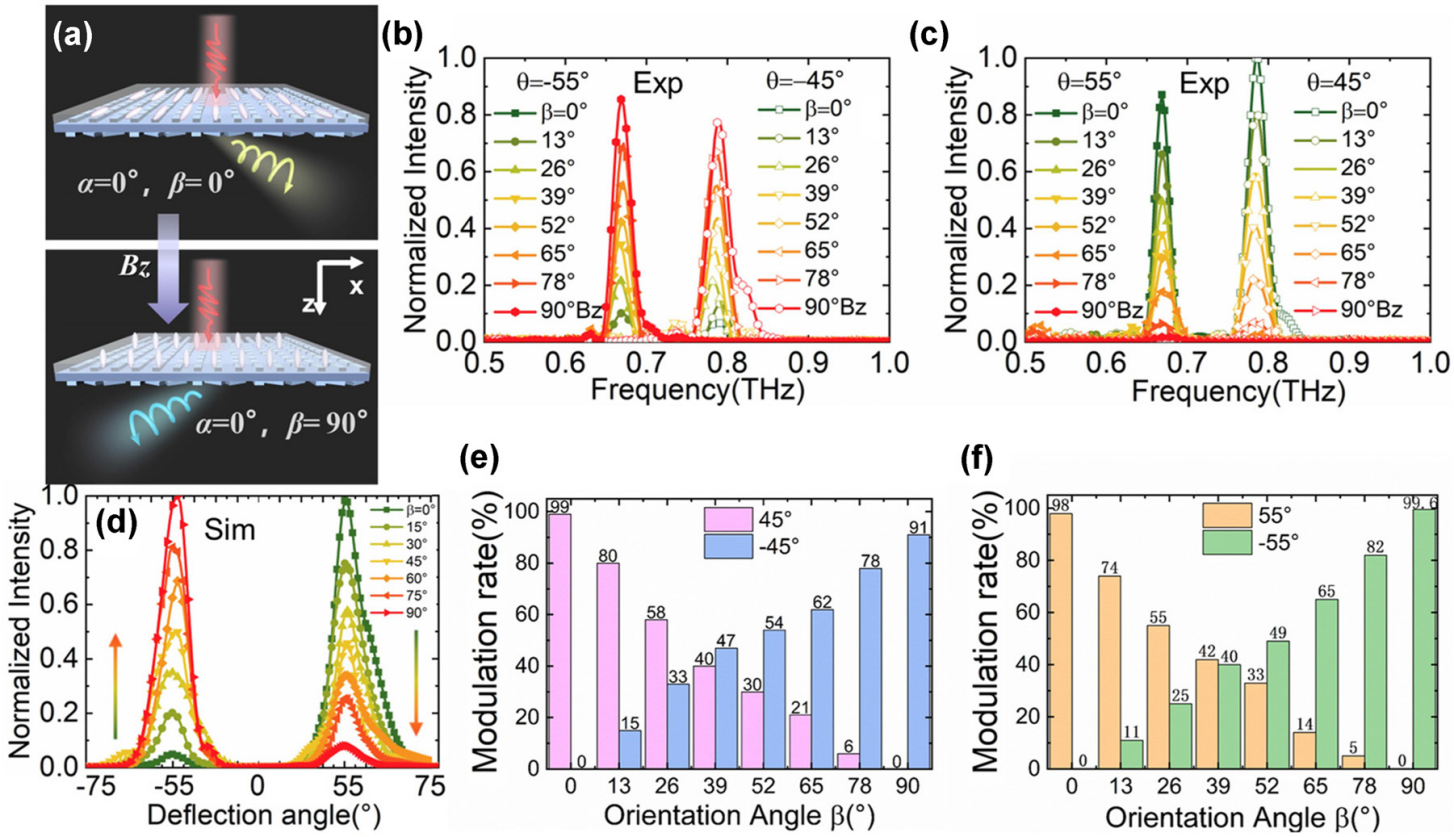
Dynamic energy distribution results with a longitudinal magnetic field. (a) Schematic diagram of 45°-LP light passing through the metadevice, where the LC orientation shifts from the *y*-axis to the *z*-axis (orientation angle *β* from 0° to 90°) as the longitudinal EMF increases. The experimental THz spectra at (b) deflection angles *θ* of −45° and −55° and (c) 45° and 55°; (d) the simulation of diffraction angle spectra in the ±1st order at 0.68 THz. Experimental modulation rate with the LC orientation angle *β* at deflection angle (e) ±45° and (f) ±55°.

Next, to investigate the dynamic energy distribution process of the device, we analyze the results with the increase of the EMF. Since the relationship between the EMF and the director angle is nonlinear, to analyze conveniently, we use the orientation angles of LC (*α* and *β*) to describe the dynamic response of the device under the different EMFs. First, we applied the longitudinal EMF to make the LC rotate in the *y*–*z* plane. When *α* = 0°, the correspondence between the EMF and the orientation angle *β* of the LC is shown in Table S2 of Supplementary Material. In this case, the orientation angle *β* changes from 0 to 90°, and the birefringence phase shift of the LC anisotropic metasurface is gradually converted from 270° to 90°. The polarization state of the input LP light is initially converted to the *L* state and gradually becomes more *R*-spin component as the EMF increases. The *L* and *R* states are separated into positive and negative angles in space after passing through the PB metasurface layer, respectively, and the proportion of energy distribution between the two sides is consistent with the proportion between the *L* and *R* spin component, as shown in Figure 5(a). Therefore, the proportion of energy distribution can be actively tuned by the orientation angle *β*. As shown in Figure 5(b) and (c), as the EMF increases, the output beam gradually decreases at the positive deflection angle and increases at the negative deflection angle. In addition, the simulation of diffraction angle spectra in the ±1st order at 0.68 THz (i.e. the corresponding central angle of 55°) is also obtained as shown in Figure 5(d). As the LC orientation shifts from the *y*-axis to the *z*-axis, the output beam gradually increases at this angle, which is consistent with the experimental results in Figure 5(b).

To visualize the dynamic modulation process of the device more intuitively, we discuss the modulation rate of the output beam at different deflection angles with the increase of EMF, which follows *M* = (*I*_B_ − *I*_0_)/max{*I*_B_, *I*_0_} where *I*_B_ is the intensity transmittance at the different EMF, and *I*_0_ is the intensity transmittance without the EMF. Figure 5(e) and (f) show the modulation rate at a deflection angle of ±45° and ±55° with the changes in the LC orientation angle from 0 to 90°, respectively, and the maximum modulation rate can reach 99 %. It can be seen that the modulation rate is always monotonically increasing or decreasing for the positive and negative deflection angles.

### 3.4 Dynamic energy distribution with a transverse magnetic field

**Figure 6:**
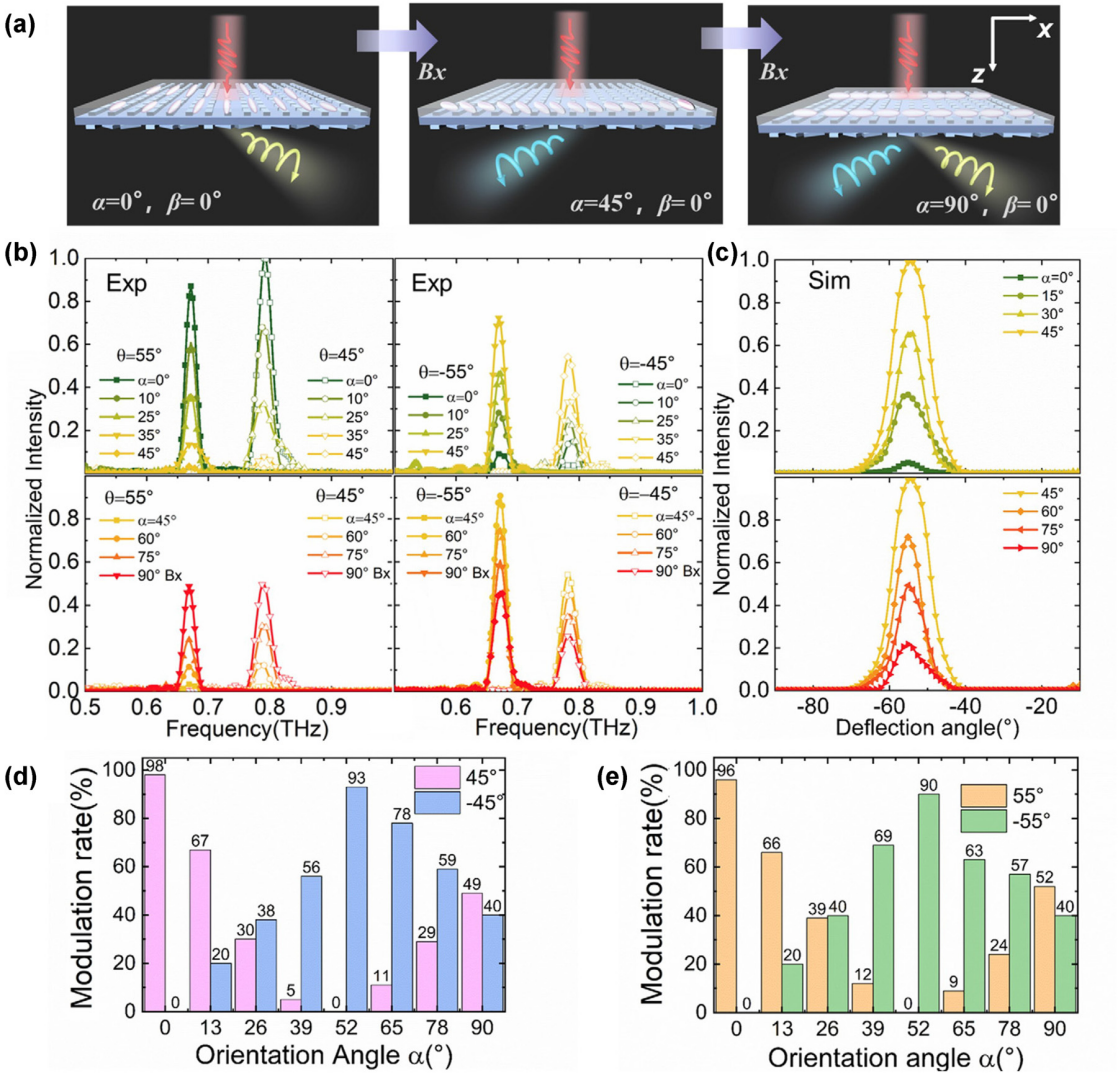
Dynamic energy distribution results with a transverse magnetic field. (a) Schematic diagram of 45°-LP light passing through the metadevice, where the LC orientation shifts from the *y*-axis to the *x*-axis (orientation angle *α* from 0° to 90°) as the transverse EMF increases. (b) The experimental THz spectra at ±45° and ±55°; (c) the simulation of diffraction angle spectra in the −1st order at 0.68 THz. Experimental modulation rate with the LC orientation angle *α* at deflection angle (d) ±45° and (e) ±55°.

When the EMF is applied along the *x*-axis, the LC anisotropic metasurface undergoes a different dynamic modulation process. When *β* = 0°, the correspondence between the EMF and the orientation angle *α* of the LC molecules is shown in Table S1 of Supplementary Material. As shown in Figure 6(a), when the orientation angle *α* turns from 0 to 90° and a 45° LP state is incident, the polarization state conversion experiences two processes: from 0 to 45°, the anisotropic phase shift is tuned from 270° to 90°, at this time the output polarization gradually changes from *L* state to *R* state; from 45° to 90°, the anisotropy changes from 90° to 0° as shown in Section S6 of Supplementary Material, and the output polarization state gradually changes from *R* state to *LP* state. The *L* and *R* spin components are separated after passing through the PB metasurface layer as shown in Figure 6(a), so that the energy distribution also expresses two processes, rather than simply shifting from the right side to the equal double sides. The outgoing beam is deflected to the right side with the *L* state first, then to the left side with the *R* state, and finally to the double sides with equal energy.

The results of the experimental THz spectra at *θ* = ±45° and ±55° shown in Figure 6(b) fully confirm the above analysis, and the simulation of the diffraction angle spectra at 0.68 THz shown in Figure 6(c) is also in agreement with the analysis and the experiment. They show that the energy at *θ* > 0 decreases from the maximum to 0, and then rises to the middle value; the energy of *θ* < 0 rises from the minimum to the highest, and then falls back to the middle, close to the positive angle. In the same way, we also obtain the modulation rate with the changes in the orientation angle *α* as shown in Figure 6(d) and (e). For *θ* = 45°, the modulation rate decreases from the maximum of 99 % to 0 and then increases to 49 %. For *θ* = −45°, the modulation rate increases from 0 to 93 %, and finally decreases from 40 %. The results of 55° are similar to that of 45°. Therefore, the modulation process of the device is more complex when the transverse EMF is applied, and the functions are richer than that of the longitudinal one, which is due to the different anisotropy and chiral asymmetric transmission in the two cases.

## 4 Conclusions

In summary, we demonstrate the LC cascaded bilayer metasurface that includes the LC layer, anisotropic metasurface, and PB metasurface. By controlling the anisotropy and chirality effects originating from both LC orientation and metasurface, dynamic spin asymmetric transmission is realized with different polarization conversions. Meanwhile, depending on the different orientations of the LC molecules under the external field, two different dynamic energy distribution processes are realized between the positive and negative deflection angles corresponding to the *L* and *R* states. The modulation rates of the two active modulation processes reach 99 % and 98 %, respectively. The beam can be deflected in a wide scanning range of ±35° to ±75° corresponding to 0.6–1.1 THz, and the maximum proportion of energy distribution when the energy is distributed on the left and right sides can reach −26 dB. This work shows that tunable anisotropy leads to dynamic modulation of asymmetric deflection in LC cascaded metasurfaces, and leads to more functionality than traditional spin asymmetric wavefront manipulation, providing a new method for multi-functional THz spin separation, conversion, and efficient large-angle beam scanning, with important potentials in wavelength/polarization division multiplexing and frequency-scanning antenna for large-capacity THz wireless communication, radar, and imaging systems.

## Supplementary Material

Supplementary Material Details

## References

[1] Sengupta K., Nagatsuma T., Mittleman D. M. (2018). Terahertz spectroscopy and imaging – modern techniques and applications. *Nat. Electron.*.

[2] Zhou R. Y., Wang C., Xu W. D., Xie L. J. (2019). Biological applications of terahertz technology based on nanomaterials and nanostructures. *Nanoscale*.

[3] Jepsen P. U., Cooke D. G., Koch M. (2011). Terahertz spectroscopy and imaging – modern techniques and applications. *Laser Photon. Rev.*.

[4] Zhang Z. Y., Zhong C. Z., Fan F., Liu G. H., Chang S. J. (2021). Terahertz polarization and chirality sensing for amino acid solution based on chiral metasurface sensor. *Sens. Actuators, B*.

[5] Gao Y., Kaushik S., Philip E. J. (2020). Chiral terahertz wave emission from the Weyl semimetal TaAs. *Nat. Commun*..

[6] Karimi E., Marrucci L., Grillo V., Santamato E. (2012). Spin-to-orbital angular momentum conversion and spin-polarization filtering in electron beams. *Phys. Rev. Lett.*.

[7] Zhao H. J., Fan F., Ji Y. Y., Jiang S. L., Tan Z. Y., Chang S. J. (2022). Active terahertz beam manipulation with photonic spin conversion based on a liquid crystal Pancharatnam-Berry metadevice. *Photonics Res.*.

[8] Deng L. G., Li Z. L., Zhou Z. (2022). Bilayer-metasurface design, fabrication, and functionalization for full-space light manipulation. *Adv. Opt. Mater*..

[9] Wang Z. X., Wu J. W., Wu L. W. (2021). High efficiency polarization-encoded holograms with ultrathin bilayer spin-decoupled information metasurfaces. *Adv. Opt. Mater*..

[10] Zhang X. Q., Yang S. M., Yue W. S. (2019). Direct polarization measurement using a multiplexed Pancharatnam-Berry metahologram. *Optica*.

[11] Jia M., Wang Z., Li H. T. (2019). Efficient manipulations of circularly polarized terahertz waves with transmissive metasurfaces. *Light: Sci. Appl.*.

[12] Tymchenko M., Gomez-Diaz J. S., Lee J., Nookala N., Belkin M. A., Alu A. (2015). Gradient nonlinear pancharatnam-Berry metasurfaces. *Phys. Rev. Lett.*.

[13] Zhu W. G., Zheng H. D., Zhong Y. C., Yu J. H., Chen Z. (2021). Wave-vector-varying Pancharatnam-Berry phase photonic spin Hall effect. *Phys. Rev. Lett.*.

[14] Cong L. Q., Xu N. N., Han J. G., Zhang W. L., Singh R. (2015). A tunable dispersion-free terahertz metadevice with Pancharatnam-Berry-phase-enabled modulation and polarization control. *Adv. Mater*..

[15] Liu J. Y., Zhang T. R., Tan Z. Y., Cheng J. R., Chang S. J., Fan F. (2023). Chiral enantiomer recognition of amino acids enhanced by terahertz spin beam separation based on a Pancharatnam-Berry metasurface. *Opt. Lett*..

[16] Zhang L., Liu S., Li L. L., Cui T. J. (2017). Spin-controlled multiple pencil beams and vortex beams with different polarizations generated by Pancharatnam-Berry coding metasurfaces. *ACS Appl. Mater. Interfaces*.

[17] Wang Z., Dong S. H., Luo W. J. (2018). High-efficiency generation of Bessel beams with transmissive metasurfaces. *Appl. Phys. Lett*..

[18] Avayu O., Almeida E., Prior Y., Ellenbogen T. (2017). Composite functional metasurfaces for multispectral achromatic optics. *Nat. Commun*..

[19] Chen Y., Yang X. D., Gao J. (2019). 3D Janus plasmonic helical nanoapertures for polarization-encrypted data storage. *Light: Sci. Appl.*.

[20] Yao B. S., Zang X. F., Li Z. (2020). Dual-layered metasurfaces for asymmetric focusing. *Photonics Res.*.

[21] Cheng J. R., Yang Y., Chen S. (2023). Continuous terahertz omnidirectional beam steering by dual diffraction of metagratings. *Photonics Res.*.

[22] Mansouree M., Kwon H., Arbabi E., McClung A., Faraon A., Arbabi A. (2020). Multifunctional 2.5D metastructures enabled by adjoint optimization. *Optica*.

[23] Bao Y. J., Nan F., Yan J. H., Yang X. G., Qiu C. W., Li B. J. (2022). Observation of full-parameter Jones matrix in bilayer metasurface. *Nat. Commun*..

[24] Xie R. S., Zhai G. H., Wang X. (2019). High-efficiency ultrathin dual-wavelength Pancharatnam-Berry metasurfaces with complete independent phase control. *Adv. Opt. Mater*..

[25] Zhang S., Zhou J. F., Park Y. S. (2012). Photoinduced handedness switching in terahertz chiral metamolecules. *Nat. Commun*..

[26] Kindness S. J., Almond N. W., Michailow W. (2020). A terahertz chiral metamaterial modulator. *Adv. Opt. Mater*..

[27] Li J. T., Li J., Zheng C. L. (2021). Dynamic control of reflective chiral terahertz metasurface with a new application developing in full grayscale near field imaging. *Carbon*.

[28] Li W. L., Hu X. M., Wu J. B. (2022). Dual-color terahertz spatial light modulator for single-pixel imaging. *Light: Sci. Appl.*.

[29] Sun Y., Xu Y., Li H. L. (2022). Flexible control of broadband polarization in a spintronic terahertz emitter integrated with liquid crystal and metasurface. *ACS Appl. Mater. Interfaces*.

[30] Dolan J. A., Cai H. G., Delalande L. (2021). Broadband liquid crystal tunable metasurfaces in the visible: liquid crystal inhomogeneities across the metasurface parameter space. *ACS Photonics*.

[31] Shen Z. X., Zhou S. H., Li X. A. (2020). Liquid crystal integrated metalens with tunable chromatic aberration. *Adv. Photonics*.

[32] Zhuang X. L., Zhang W., Wang K. M. (2023). Active terahertz beam steering based on mechanical deformation of liquid crystal elastomer metasurface. *Light: Sci. Appl.*.

[33] Komar A., Paniagua-Dominguez R., Miroshnichenko A. (2018). Dynamic beam switching by liquid crystal tunable dielectric metasurfaces. *ACS Photonics*.

[34] Naveed M. A., Kim J., Javed I. (2022). Novel spin-decoupling strategy in liquid crystal-integrated metasurfaces for interactive metadisplays. *Adv. Opt. Mater*..

[35] Isic G., Vasic B., Zografopoulos D. C., Beccherelli R., Gajic R. (2015). Electrically tunable critically coupled terahertz metamaterial absorber based on nematic liquid crystals. *Phys. Rev. Appl*..

[36] Chen C. C., Chiang W. F., Tsai M. C. (2015). Continuously tunable and fast-response terahertz metamaterials using in-plane-switching dual-frequency liquid crystal cells. *Opt. Lett*..

[37] Ung B. S. Y., Liu X. D., Parrott E. P. J. (2018). Towards a rapid terahertz liquid crystal phase shifter: terahertz in-plane and terahertz out-plane (TIP-TOP) switching. *IEEE Trans. Terahertz Sci. Technol.*.

