# Supplementary material for: Broadband large-angle beam scanning with dynamic spin energy distribution based on liquid crystal cascaded bilayer metasurface
Source: Nanophotonics. 2023 Sep 29;12(20):3945–54. doi: 10.1515/nanoph-2023-0468 (PMC11501543; doi:10.1515/nanoph-2023-0468)
Supplement: Supplementary file 1 — Supplementary Material Details [file j_nanoph-2023-0468_suppl_001.docx]

**Supplementary material:**

**Broadband large-angle beam scanning with dynamic spin energy distribution based on liquid crystal cascaded bilayer metasurface**

Huijun Zhao ^a^, Jiayue Liu^a^, Songlin Jiang^a^, Xinhao Jiang^a^, Jierong Cheng^a^, Yunyun Ji ^a, b^, Shengjiang Chang ^a, b^ , Fei Fan ^a, b, *^

*^a^ Institute of Modern Optics, Nankai University, Tianjin Key Laboratory of Micro-scale Optical Information Science and Technology, Tianjin 300350, China*

*^b^ Tianjin Key Laboratory of Optoelectronic Sensor and Sensing Network Technology, Tianjin 300350, China*

****Corresponding author:* [*fanfei@nankai.edu.cn*](mailto:fanfei@nankai.edu.cn)

**Contents**

1. Experimental methods
2. Experimental THz spectra in different magnetic field directions
3. Numerical simulation method
4. Design of the PB metasurface
5. Simulation of the modulation process under a longitudinal magnetic field
6. Simulation design of LC anisotropic metasurface under a transverse magnetic field
7. Simulation of the modulation process under a transverse magnetic field
8. **Experimental methods**

We built an angle-resolved terahertz (THz) time-domain spectroscopy (AR-THz-TDS) system to measure the spin conversion and energy distribution of this composite metadevice, as shown in Figure 1b. THz pulses are generated by a GaAs photoconductive antenna (PCA) that is excited by a fiber-optic femtosecond laser at 780nm for a duration of 80fs. The sample is placed in the center of a rotating table and a 2 cm diameter collimated THz beam is incident onto the sample. The other PCA is fiber-coupled through signal amplification and phase-lock, acting as a THz detector, which is placed in a rotating stage to rotate the deflection angle *θ* for angle-resolved time-domain signal measurements. All experiments are carried out at room temperature (20 ± 2°C) and relative humidity <30%. Two THz metal wire polarizers are placed before and after the sample to generate and detect quadrature polarization states, which have nearly 100% transmittance and more than 99.8% polarization degree in the THz regime. In addition, to control the orientation of the LC molecules, two variable magnets placed to the left and right of the sample are used to generate a transverse EMF of 0-60 mT. Two variable magnets on the front and back sides of the sample are used to generate a longitudinal EMF of 0-60mT.

To simulate the transmission spectra of the PB metasurface, a pair of orthogonal polarization states need to be input.

For circularly polarized (CP) basis vectors, the output is two converted (*t_rl_* and *t_lr_*) and two unconverted (*t_rr_* and *t_ll_*) states. The first subscript indicates the polarization state of the output component; The second component represents the polarization state of the input wave. To obtain these four CP states, we can rotate the two THz polarizers to ±45° in the THz time-domain polarization spectroscopy system to measure the four linear co- and cross-polarization transmission coefficients *t_++45°_*, *t_+−45°_*, *t_−+45°_*, and *t_−−45°_*, respectively. The transmission matrix T of a chiral device is given by the following equation

 (S1)

For linearly-polarized light base vector, the intensity transmittance of the output y-LP light is I=(A_sam_/A_ref_)^2^, where A_sam_ is the amplitude value of the measured sample's time domain signal after Fourier transform, and A_ref_ is the amplitude value of the measured air-referenced time domain signal after Fourier transform.

To detect the arbitrary polarization state of the emitted light, we can rotate the second THz polarizer to ±45° to obtain the transmission amplitudes *A_+45°_*, *A_−45°_* and phase *φ_+45°_* , *φ_-45°_* for the ±45° linearly polarization (LP) component. Thus, for the incidence of the 45°-LP state, the transmission of *L* and *R*-states of the output is given by the following equation:

$\begin{matrix} I_{L}\left(\omega\right)=\left(\frac{A_{+45^{\circ}}\left(\omega\right)e^{i\sigma_{+45^{\circ}}\left(\omega\right)}-iA_{-45^{\circ}}\left(\omega\right)e^{i\sigma_{-45^{\circ}}\left(\omega\right)}}{2}\right)^{2} \\ I_{R}\left(\omega\right)=\left(\frac{A_{+45^{\circ}}\left(\omega\right)e^{i\sigma_{+45^{\circ}}\left(\omega\right)}+iA_{-45^{\circ}}\left(\omega\right)e^{i\sigma_{-45^{\circ}}\left(\omega\right)}}{2}\right)^{2} \end{matrix}$ (S2)

The transmission results shown in the text are all intensity transmission spectra, that is, *I_LL_*=*t_ll_^2^*, *I_RR_*=*t_rr_^2^*, *I_LR_*=*t_lr_^2^* and *I_RL_*=*t_rl_^2^.*

To intuitively describe the arbitrary polarization state of the output light, we can calculate the terminal trajectory equation of electric vector *E*, that is, polarization ellipse, as follows:

$\left(\frac{E_{x}}{A_{-45^{\circ}}}\right)^{2}+\left(\frac{E_{y}}{A_{+45^{\circ}}}\right)^{2}-\frac{2E_{x}E_{y}}{A_{-45^{\circ}}A_{+45^{\circ}}}\cos\Delta\delta=\sin^{2}\Delta\delta$ (S3)

where the phase difference *Δδ*=*δ*_+45°_-*δ*_-45°._

In addition, when the EMF is applied in different directions, the polarization conversion and chiral modulation process of the device are different. To discuss the ellipticity (*ε*) and CD spectra of one supercell varying with the orientation angle and the frequency, the equations are as follows:

$\varepsilon=I_{L}(dB)-I_{R}(dB)$ (S4)

$CD=\frac{I_{\text{LL}}-I_{RR}}{I_{\text{LL}}+I_{RR}}\times100\%$ (S5)

where *I_LL_* and *I_RR_* are the intensity transmittance (in %), and *I_L_* and *I_R_* are the intensity transmission (in dB) of the device.

The relationship between the orientation angle (*α* & *β*) and the EMF is determined by the experimental data. Since the relationship between the EMF and the director angle is nonlinear, to analyze conveniently, we use the changes in the orientation angle to describe the dynamic optical response of LCs. The anisotropic phase shift values under different magnetic fields have been measured, and the orientation angle of the LC can be calculated by the theoretical formula as follows:

When no magnetic field is applied, the LC molecules are arranged in the direction of the *y*-axis and the angle between the director angle of LCs and *y*-axis is defined as *α* = 0°. A transverse magnetic field is gradually applied along the *x*-axis, and the orientation angle *α* gradually increases from 0° to 90°. The LCs are modeled as a uniaxial model, and their refractive index is given by the tensor [1, 2]

$n_{LC}=\left(\begin{matrix} n_{xx}=n_{effx} & 0 & 0 \\ 0 & n_{yy}=n_{effy} & 0 \\ 0 & 0 & n_{zz}=n_{o} \end{matrix}\right)$ (S6)

$n_{effx}(\alpha)=n_{o}n_{e}/\sqrt{n_{o}^{2}\cos^{2}\alpha+n_{e}^{2}\sin^{2}\alpha}$ (S7)

$n_{effy}(\alpha)=n_{o}n_{e}/\sqrt{n_{o}^{2}\sin^{2}\alpha+n_{e}^{2}\cos^{2}\alpha}$ (S8)

The phase difference *φ_yx_* of the LC between two orthogonal polarization components can be calculated by

 (S9)

where *c* is the speed of light in vacuum, *f* is the frequency, *d_LC_* is the thickness of LCs, *n_effx_* and *n_effy_* are the refractive indices of the *x* components and *y* components, which are the function of *α*. In the experiment, a phase shift value can be obtained under each magnetic field, so that the relationship between the magnetic field and the orientation angle *α* can be obtained by (S6) ~ (S9).The results are shown in Table S1.

When a longitudinal magnetic field is gradually applied along the *z*-axis, we define the angle between the LC orientation and the x-y plane as *β*, the refractive index tensor of LC is shown below

$n_{LC}=\left(\begin{matrix} n_{xx}=n_{o} & 0 & 0 \\ 0 & n_{yy}=n_{effy} & 0 \\ 0 & 0 & n_{zz}=n_{effz} \end{matrix}\right)$ (S10)

$n_{effy}(\beta)=n_{o}n_{e}/\sqrt{n_{o}^{2}\sin^{2}\beta+n_{e}^{2}\cos^{2}\beta}$ (S11)

The phase difference *φ_yz_* between the two orthogonal polarized components is changed to the following equation

 (S12)

Similarly, an experimental phase shift value can be obtained for each longitudinal magnetic field, so that the relationship between the magnetic field and the orientation Angle can be obtained by (S11) ~ (S12). The results are shown in Table S2.

**Table S1:**

The correspondence between the applied EMF and the orientation angle *α* of the LC molecules when *β*=0°.

| External magnetic field(mT) | 0 | 8 | 17 | 26 | 36 | 45 | 53 | 60 |
| --- | --- | --- | --- | --- | --- | --- | --- | --- |
| Birefringence phase shift(°) | 270 | 225 | 185 | 145 | 90 | 75 | 43 | 30 |
| Orientation angle *α*(°) | 0 | 10 | 25 | 35 | 45 | 60 | 75 | 90 |

**Table S2:**

The correspondence between the applied EMF and the orientation angle *β* of the LC molecules when *α*=0°.

| External magnetic field(mT) | 0 | 8 | 17 | 26 | 36 | 45 | 53 | 60 |
| --- | --- | --- | --- | --- | --- | --- | --- | --- |
| Birefringence phase shift(°) | 270 | 245 | 218 | 192 | 167 | 141 | 115 | 90 |
| Orientation angle *β*(°) | 0 | 13 | 26 | 39 | 52 | 65 | 78 | 90 |

1. **Experimental THz spectra in different magnetic field directions**


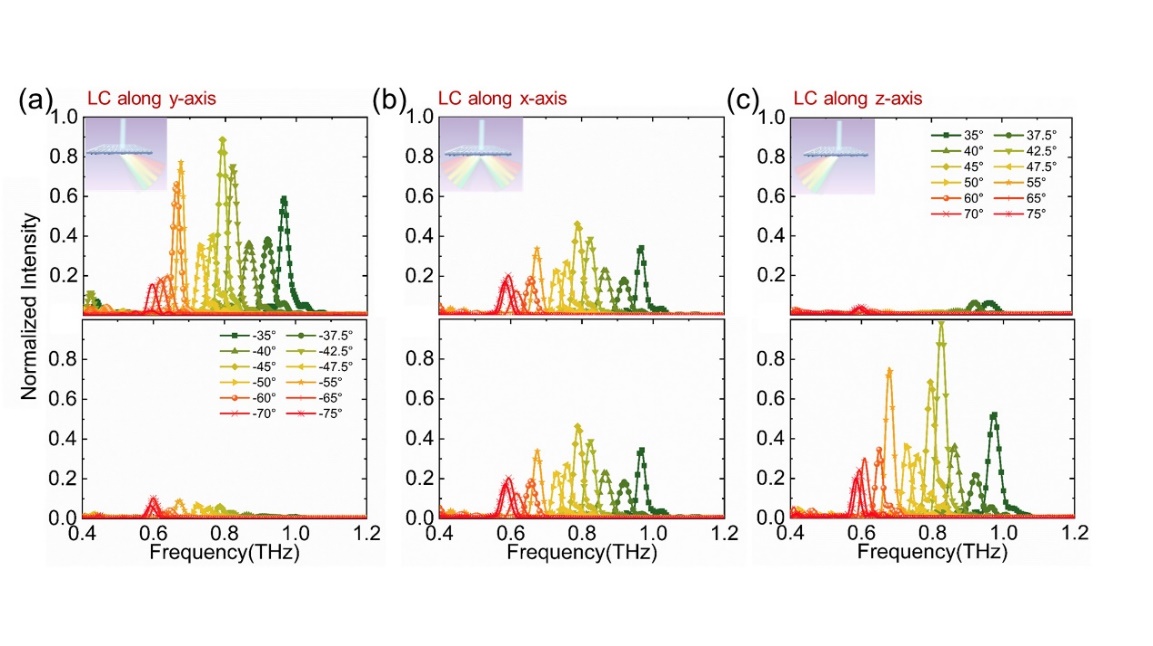


**Figures S1**. Experimental THz spectra at different deflection angles *θ* with LC orientations along *x, y,* and *z* axis under the different EMF directions: (a) no EMF is applied and the LC molecules are along the *y*-axis; (b) A transverse EMF is applied and the LC is orientated along the *x*-axis; (c) A longitudinal EMF is applied and the LC is orientated along the *z*-axis.

The experimental intensity transmittance of the device at different deflection angles is shown in Figure S1. When there is no EMF applied and the LC molecules are along the *y*-axis, some significant signals can be detected at the deflection angles *θ* from 35° to 75°, as shown in Figure S1a. The intensity transmittance at negative deflection angles is much less than 10%, so it can be assumed that there is no signal emission at these angles. When a transverse magnetic field is applied and the LC is orientated along the *x*-axis, a valid signal can be detected at both positive and negative deflection angles. When a longitudinal EMF is applied and the LC is orientated along the z-axis, the effective signal can only be detected at a negative deflection angle. Therefore, by applying EMF in different directions, two different dynamic energy distribution processes can be achieved between positive and negative deflection angles.

1. **Numerical simulation method**

We first used the finite time domain difference (FDTD) method in the commercial software of Lumerical FDTD Solutions 2020 R2 to complete the simulation design of the device, and simulate the electric field distributions and transmission spectra of the composite metadevice. Set periodic boundary conditions in the *x* and *y* directions and open boundary conditions in the *z*-direction. CP light in different spin states incident perpendicular to the metadevice requires the synthesis of two LPs with equal amplitude and 90° phase difference. The output time domain signal can be detected with a point detector. According to the electric field obtained by the point monitor, the Fourier transform is used to simulate the transmission and phase of different elements. By considering the composite anisotropic meta-atoms irradiated by a 45°-LP wave, the simulated birefringence phase shift and intensity transmission spectra are obtained. CP light is incident into three meta-atoms within a PB supercell to obtain relative phase shift spectra. The 45°-LP light is incident to the composite metadevice, the point detector is placed in different deflection directions to obtain the intensity transmission spectra in all working spectrums, and the surface detector placed in the *x*-*z* plane is used to obtain the electric field distributions. In addition, Gaussian beams with polarization along the *x*-axis (or *y*-axis) are often incident into the actual (finite-size) structure, where open boundary conditions are set on the *x*, *y*, and *z* axes. A DFT planar monitor is used to obtain the *x*-*y* plane far-field distribution matrix. The energy is concentrated at the diffraction angle corresponding to the source frequency, and finally, the angular transmission distribution spectra at that frequency are obtained.

The full-wave simulation software CST 2020 is used to simulate the far-field scattering patterns of the composite metadevice. In the simulation, open (add space) boundary conditions are set in the *x* and *y* directions, and open boundary conditions in the *z* direction, which consists of perfectly matched layers. The source is a small *z*-aligned CST discrete port that is placed 300 μm from the top of the LC. Si is set to a material with a dielectric constant of 11.69, LC is set to a uniaxial birefringence crystal, that is, the extraordinary refractive index (*n_e_*) is different from the ordinary refractive index (*n_o_*), the dielectric constant of the major and minor axes is set to 3.61 and 2.56, respectively, and the optical axis of the LC can rotate in three-dimensional space. For the far-field diffraction signal at a single frequency, to make it closer to the theoretical impulse pulse, we draw 20*20 supercell structures. Then far-field probes with different frequency points were set to detect the far-field, and these probes were placed 300μm below the PB metasurface. The far-field scattering patterns was shown in Figure 3a.

1. **Design of the PB metasurface**


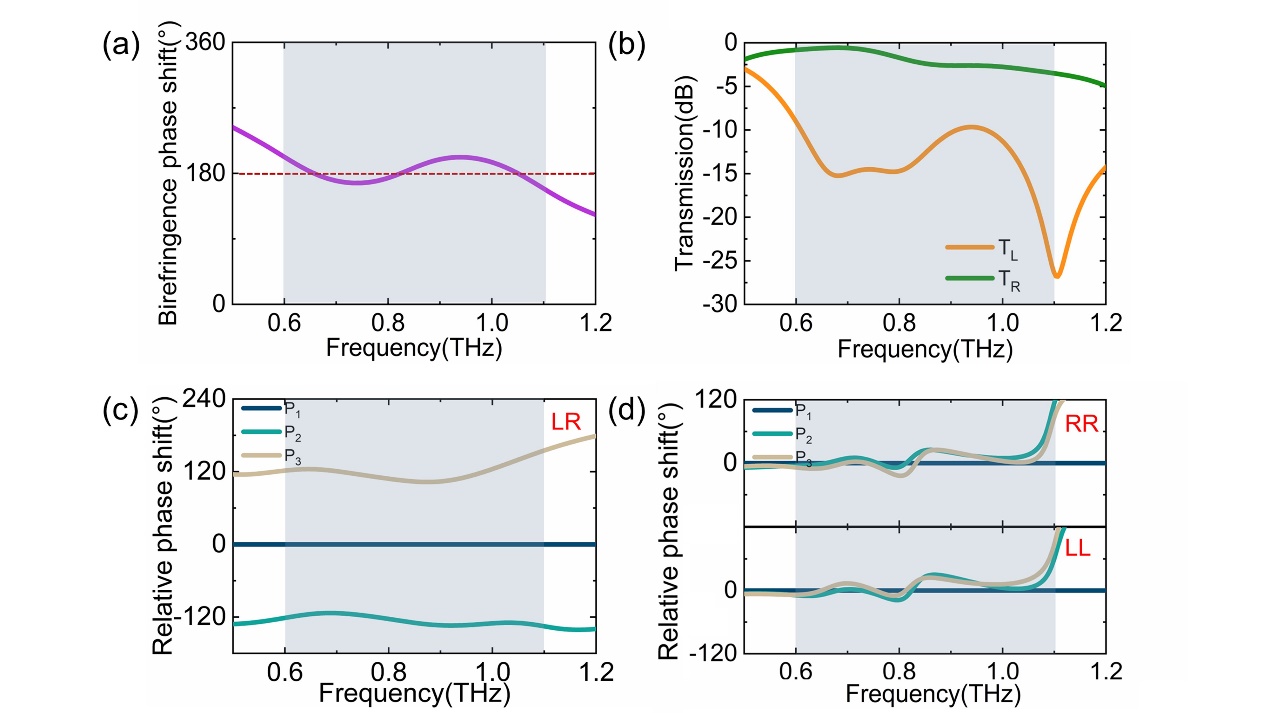


**Figure S2**. (a) Simulation birefringence phase shift of PB metasurface in the orthogonal direction of *x* and *y* axes; (b) Transmission spectra of different spin states passing through PB metasurface when *L*-state incident. Relative phase shift spectra of (c) spin-flip state *LR* and (d) spin-lock state *RR* and *LL* for the three meta-atoms (*P_1_*, *P_2,_* and *P_3_*).

Figure S2a shows the birefringence phase shift of the PB metasurface obtained by simulation in the orthogonal directions of the *x*- and *y*-axes. In the range of 0.6~1.1THz, the phase difference between the *x-* and *y*-axis directions is about 180°. At this point, the CP light passing through the PB metasurface will be converted to its spin-flipped state. As shown in Figure S2b, the output *L*-state component is always much larger than the *R*-state, and the insertion loss is less than -5dB, indicating that the *L*-state is essentially converted to the *R*-state. In addition, we obtain the relative phase shift spectra at the *R*-state incidence, as shown in Figure S2c. For spin-flip state *LR*, there is a positive spatial gradient phase distribution in the turn of *P_1_˃P_2_˃P_3_* with a step of 120° in the whole frequency range of 0.6~1.1 THz, and finally, the total phase shift is 2π in one PB supercell. The spin-lock *RR* and *LL* do not satisfy the spatial gradient phase distribution required for beam deflection, which means that their wavefront is not deflected, as shown in Figure S2d.

1. **Simulation of the modulation process under a longitudinal magnetic field**

**
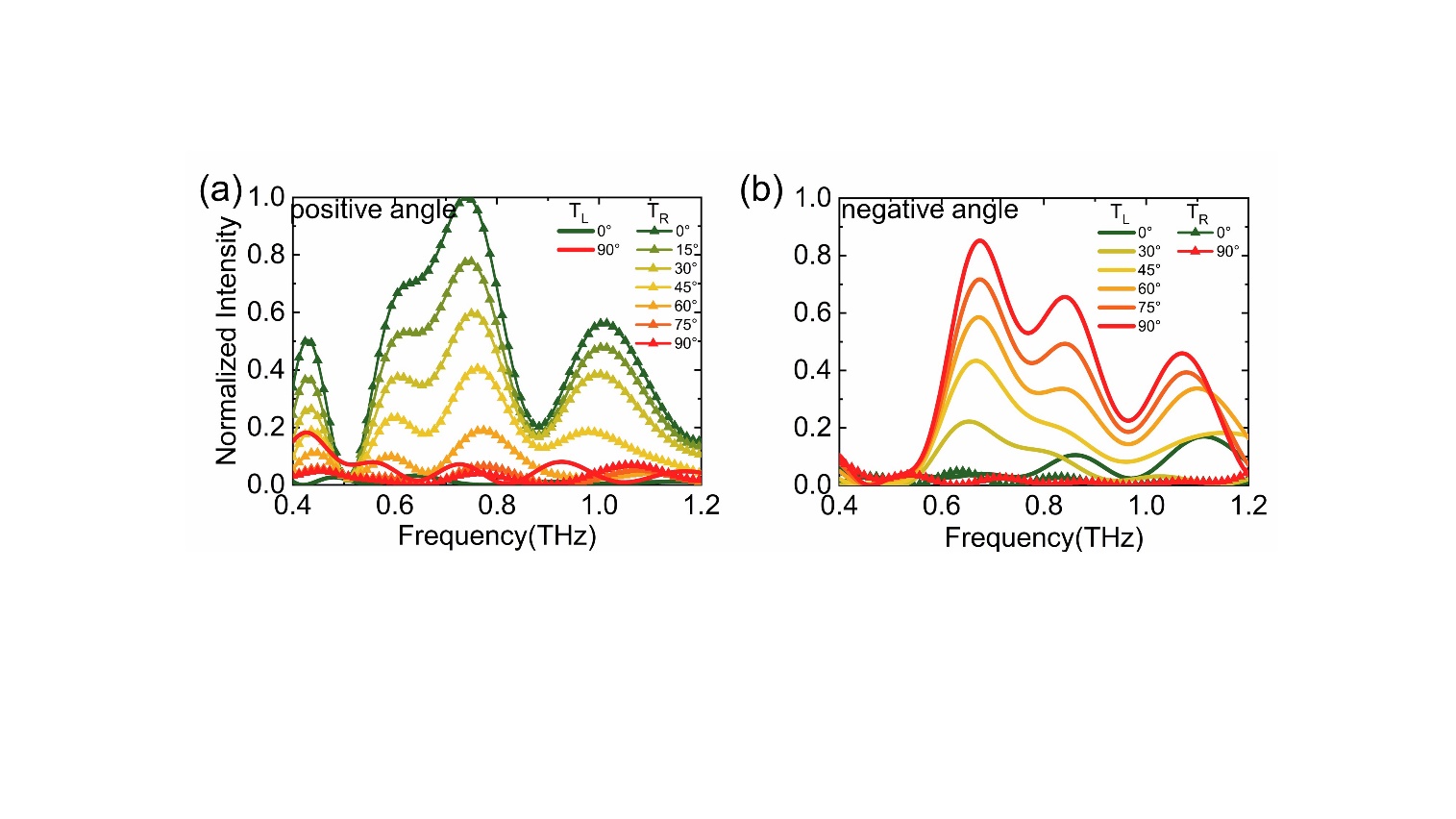
**

**Figure S3.** Near-field spectra of the different spin states of the device at the (a) positive and (b) negative angles as the LC orientation shifts from the *y*-axis to the *z*-axis (orientation angle *β* from 0°-90°).

Next, we placed the detector at 2500um behind the sample to obtain the simulated near-field spectra of the device, as shown in Figure S3, the dynamic modulation process of the device with a longitudinal rotation (*y*-*z*) of the LC orientation in different deflection directions is obtained, and the detected signal at this time is a signal in the full frequency domain and full deflection angle. As shown in Figure S3, when the LC orientation is along the *y*-axis, the device deflects the beam at a positive deflection angle. As shown in Figure S3a, b, the transmission of the *R*-state is very high, while the transmission of the *L*-state is < 0.1 and negligible. As the LC orientation gradually shifts from the *y*-axis to the *z*-axis, the peak transmission of the output *R*-state at the positive angle gradually decreases, while the peak transmission of the output *L*-state at the negative angle gradually increases. When the LC orientation is completely along the *z*-axis, the beam is completely deflected at negative angles.

1. **Design of LC anisotropic metasurface under a transverse magnetic field**

**
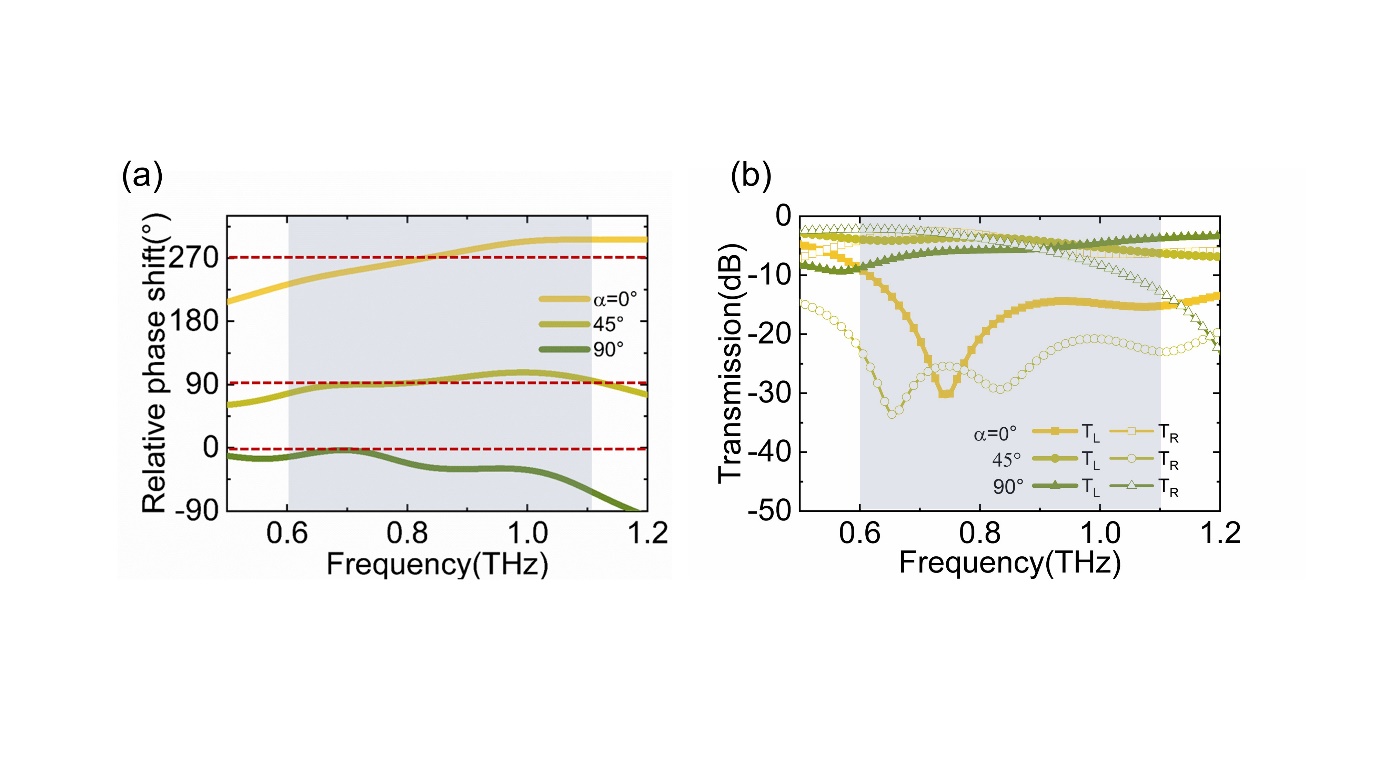
**

**Figure S4.** With the increase of transverse EMF and the LC orientation shifts from the *y*-axis to the *x*-axis (orientation angle *α* from 0°-90°). (a) Simulated birefringence phase shift of the composite anisotropic metasurface in the *x*- and *y*-axis directions; (b) Transmission spectra of the output spin state of the composite anisotropic metasurface when the LP wave is input in the polarization direction of +45°.

To analyze the dynamic energy distribution process when a transverse EMF is applied, we simulate the dynamic anisotropy and spin conversion of the composite anisotropic metasurface. As shown in Figure S4a, when LC orientation is along the *y-*axis, i.e. α=0°, the birefringence phase shift value is about 270°. Thus, when 45°-LP light is incident on this composite anisotropic metasurface, the *R*-state of the outgoing beam is always much larger than the *L*-state, with an insertion loss of less than -10dB, as shown by the yellow line in Figure S4b. Therefore, the composite anisotropic metasurface will realize the conversion of LP light to the *R*-state. As the LC orientation rotates from the *y*-axis to the *x*-axis, the birefringence phase shift changes. When the axis of the LC is in the *x*-*y* plane and the angle with the *y*-axis is 45°, i.e. α=45°, the birefringence phase shift is converted to 90°, and the output beam is converted to the *L*-state. When the LC orientation is along the *x*-axis, the birefringence phase shift is 0°, so there are both the *L* and *R* states in the outgoing beam.

1. **Simulation of the modulation process under a transverse magnetic field**

**
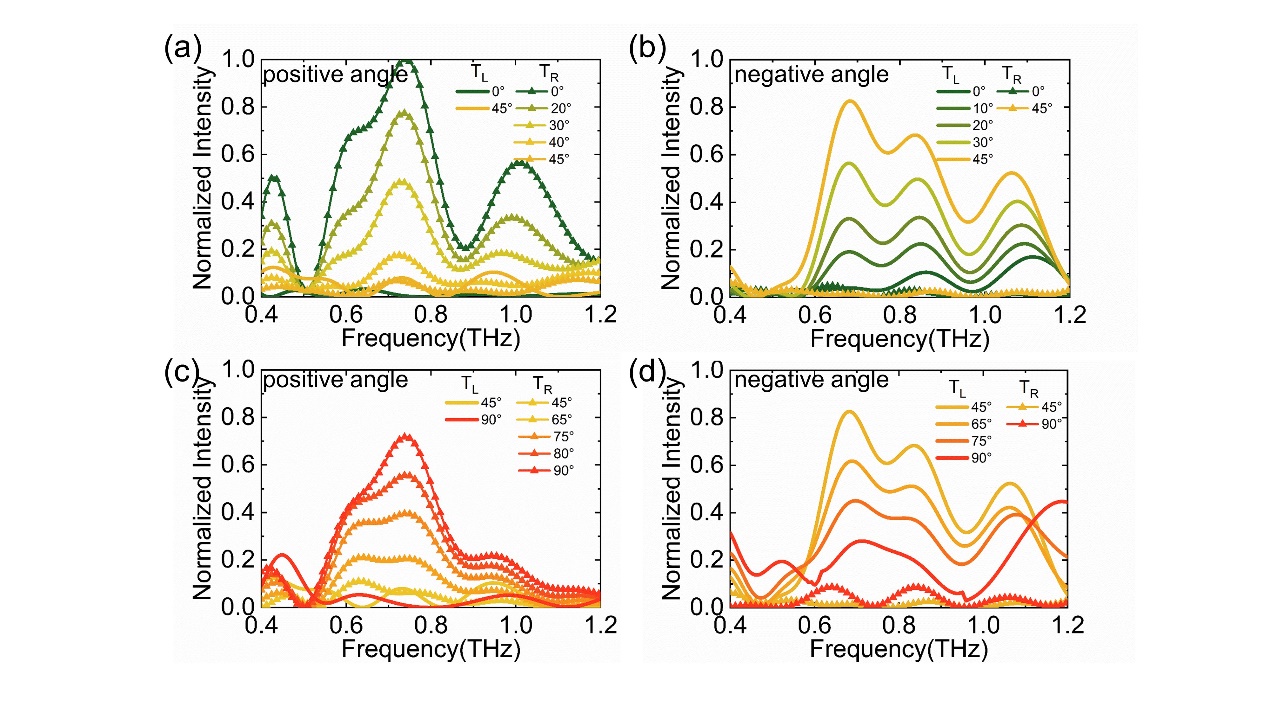
**

**Figure S5**. The LC orientation rotates within the y-x plane. When the orientation angle α turns from 0 to 45°, the near-field spectra of the device are in different spin states of (a) positive and (b) negative deflection angles. When the orientation angle α turns from 45 to 90°, the near-field spectra of the device under different spin states at (c) positive and (d) negative deflection angles.

Next, we obtained the dynamic modulation process of the device when the LC orientation shifts from the *y*-axis to the *x-*axis as the transverse EMF increases, and the detected signal at this time is a signal in the full frequency domain and full deflection angle. As shown in Figure S5, when the LC orientation is along the *y*-axis, the outgoing beam of the device is converted to the *R*-state and the deflection direction is at positive angles. As the LC rotates within the *y*-*x* plane when the LC orientation is located between the *y*-axis and *x*-axis, *i.e. α*=45°, the outgoing beam is converted into *L*-state and the deflection direction is in the negative angle direction. When the LC orientation is along the *x*-axis, the outgoing beam is converted into LP light synthesized by *L*-state and *R*-state, so there is a beam deflection output at both positive and negative deflection angles.

References

[1] L. Wang, X.W. Lin, W. Hu, G.H. Shao, P. Chen, L.J. Liang, B.B. Jin, et al. " Broadband tunable liquid crystal terahertz waveplates driven with porous graphene electrodes," *Light-Sci Appl*, vol. 4, p. e253, 2015.

[2] Y.Y. Ji, F. Fan, X.H. Wang, S.J. Chang, "Broadband controllable terahertz quarter-wave plate based on graphene gratings with liquid crystals," Opt Express, vol. 26, pp. 12852-12862, 2018.
